# CyTOF Analysis Reveals a Distinct Immunosuppressive Microenvironment in IDH Mutant Anaplastic Gliomas

**DOI:** 10.3389/fonc.2020.560211

**Published:** 2021-02-04

**Authors:** Weilun Fu, Wenjing Wang, Hao Li, Yuming Jiao, Jiancong Weng, Ran Huo, Zihan Yan, Jie Wang, Hongyuan Xu, Shuo Wang, Jiangfei Wang, Dexi Chen, Yong Cao, Jizong Zhao

**Affiliations:** ^1^ Department of Neurosurgery, Beijing Tiantan Hospital, Capital Medical University, Beijing, China; ^2^ China National Clinical Research Center for Neurological Diseases, Beijing, China; ^3^ Beijing Institute of Hepatology, Beijing YouAn Hospital, Capital Medical University, Beijing, China; ^4^ Organ Transplantation Center, The Affiliated Hospital of Qingdao University, Qingdao, China

**Keywords:** anaplastic astrocytoma, anaplastic oligodendroglioma, CyTOF/mass cytometry, immune profiling, microenvironment, glioma

## Abstract

The immune microenvironment is important for the development, progression, and prognosis of anaplastic glioma (AG). This complex milieu has not been fully elucidated, and a high-dimensional analysis is urgently required. Utilizing mass cytometry (CyTOF), we performed an analysis of immune cells from 5 patients with anaplastic astrocytoma, IDH-mutant (AAmut) and 10 patients with anaplastic oligodendroglioma, IDH-mutant and 1p/19q codeletion (AOD) and their paired peripheral blood mononuclear cells (PBMCs). Based on a panel of 33 biomarkers, we demonstrated the tumor-driven immune changes in the AG immune microenvironment. Our study confirmed that mononuclear phagocytes and T cells are the most abundant immunocytes in the AG immune microenvironment. Glioma-associated microglia/macrophages in both AAmut and AOD samples showed highly immunosuppressive characteristics. Compared to those in the PBMCs, the ratios of immune checkpoint-positive exhausted CD4+ T cells and CD8+ T cells were higher at the AG tumor sites. The AAmut immune milieu exhibits more immunosuppressive characteristics than that in AOD.

## Introduction

WHO grade III anaplastic gliomas (AGs) comprise approximately 6 to 10% of all newly diagnosed adult primary brain tumors (1). Previously, based on morphological criteria, AGs were classified into three groups: a) anaplastic oligodendroglioma, b) anaplastic oligoastrocytoma, and c) anaplastic astrocytoma. Anaplastic oligoastrocytoma accounts for 30–50% of all AGs, and the remaining 50–70% are referred to as anaplastic astrocytoma (2). In 2016, the WHO organization defined two molecular markers for histological analysis in the diagnostic and prognostic stratification of AGs (3). Based on the mutation of isocitrate dehydrogenase (IDH) and the codeletion of chromosome 1p/19q, AGs can be divided into three main distinct subgroups: 1) anaplastic astrocytoma, IDH-mutant (AAmut); 2) anaplastic astrocytoma IDH-wild-type (AAwt); and 3) anaplastic oligodendroglioma, IDH-mutant and 1p/19q codeletion (AOD) (3). The typical treatment for AGs is maximal safe resection followed by radiation therapy or chemotherapy. Despite surgery, radiation therapy and chemotherapy, the prognosis of WHO grade III glioma is still poor. The median overall survival of AGs varies widely from 3 to 12 years (4, 5).

Iris Elens investigated the immunotherapy safety and efficacy for recurrent AGs (6). After surgical resection, 39 patients received dendritic cell vaccines loaded with autologous tumor lysates (6). Compared with that of temozolomide treatment in the literature, the median progression-free survival was not significantly different after immunotherapy, although the expected outcome of immunotherapy was more pronounced than temozolomide treatment in AGs (6). The design of immunotherapy strategies for AGs requires detailed knowledge of the immune cell landscape. To our best knowledge, tumor-driven immune changes in the milieu of AGs have seldom been reported.

Patients with AOD have a more favorable prognosis than those with AAmut, even among those with the same tumor grade (7). The immune milieu acts a pivotal part in the glioma response to treatment and the prognosis (8). The difference in the immune microenvironment between these two subgroups with different prognostic estimates remains elusive.

Immunotherapy for AG is an emergent revolution that promises the prospect of highly specific and less toxic therapy compared to conventional strategies (9). Immunotherapy generically intensifies immune cell functions and facilitates improved antitumor immunity. Therefore, a wide-ranging understanding of the AG immune milieu on a high-dimensional single-cell basis is crucially needed. In the present study, we applied mass cytometry (CyTOF) to demonstrate the tumor-driven immune changes *in situ* to capture the cellular and molecular complexities of the AG immunosuppressive milieu. We compared the difference in immune signatures between the AAmut and AOD subgroups in the AGs. Our data will help elucidate the immune microenvironment changes in AGs and promote the development of immunotherapy.

## Materials and Methods

### Anaplastic Glioma Tissue and Blood Samples Collection

From June 2018 to March 2019, we enrolled patients with WHO grade III AAmut or AOD who underwent craniotomy surgery at Beijing Tiantan Hospital (Beijing, China), and blood and tumor tissues were obtained. All these patients were diagnosed and confirmed by histopathological and molecular analysis. Before sampling, none of these enrolled patients used glucocorticoids. The current study was approved by the Institutional Review Board and Ethics Committee of Beijing Tiantan Hospital, Capital Medical University. Written informed consent was obtained from all patients.

### AG Tumor Specimen Single-Cell Preparation

After the operation, the ice-cold Dulbecco’s phosphate-buffered saline (DPBS, Sigma-Aldrich) was immediately used to wash AAmut or AOD tumor tissues. In brief, type IV collagenase (Gibco) was used to dissociate the AG specimens. Next, Dulbecco’s modified Eagle’s medium (DMEM, Sigma-Aldrich) was used to wash the specimens. After centrifugation, the specimens were filtered through a 40 µm cell strainer with DPBS and washed with red blood cell (RBC) lysis buffer (BD Biosciences). Next, DPBS was used to wash the dissociated cell suspension. Finally, the cells were resuspended in staining buffer (DPBS containing 5% fetal bovine serum; ScienCell).

### Blood Specimen Single-Cell Preparation

Peripheral blood specimens were gathered with ethylenediaminetetraacetic acid anticoagulation tubes. To remove plasma, the blood specimens were centrifuged first. Then, the blood specimens were transferred into SepMate peripheral blood mononuclear cell (PBMC) isolation tubes containing Ficoll (STEMCELL Technologies). After centrifugation, RBC lysis buffer was used to wash the cells. Finally, these cells were washed with staining buffer twice.

### CyTOF Examination

A panel of 33 antibodies was used as previously reported (10). Preconjugated antibodies were purchased from Fluidigm Company. Purified antibodies were purchased from Biolegend Company and then conjugated with metals using the Maxpar^®^ X8 Multimetal Labeling Kit (Fluidigm) according to the manufacturer’s protocol. **Supplementary Figure S1** demonstrates the list of the antibodies and reporter isotopes. In brief, the cell specimens were rewarmed quickly. Anti-CD45 antibody conjugated with 156Gd was used to stain cells from AG tissues, while anti-CD45 antibody conjugated with 89Y was used to stain cells from PBMCs. We mixed together the cells from the AG and PBMC samples and then stained the specimens with cell surface antibodies. Subsequently, the mixed specimens were permeabilized and stained with intracellular antibodies. Then 0.125 nM Intercalator-Ir (Fluidigm) diluted in phosphate-buffered saline (PBS; Sigma-Aldrich) containing 2% formaldehyde was used to wash and incubate the antibody-labeled specimens. Specimens were stored at 4°C until CyTOF examination. Before acquisition, deionized water was used to wash the specimens. The specimens were resuspended in deionized water containing a 1:20 dilution of EQ Four Element Beads (Fluidigm) at a concentration of 1 × 10^6^ cells/ml. The specimens were examined by CyTOF2 mass cytometry (Fluidigm Company).

### Mass Cytometry Data Analysis

The.fcs files of CyTOF data were uploaded and analyzed with Cytobank (www.cytobank.org). As previously described (11), based on EQ Four Element Beads, we can use the MATLAB-based normalization technique according to the bead intensities. T cells were characterized as CD45+CD3+; natural killer (NK) cells were characterized as CD45+CD3−CD16+CD56+ (8, 12); B cells were characterized as CD45+CD19+; monocytes were characterized as CD45+CD14+CD16+ (13); macrophages or microglial cells were characterized as CD45+CD11b+CD3−CD19− CD66b−CD16− (14); regulatory t cells (Tregs) were characterized as CD45+CD4+CD25+CD127− (15) and granulocytes were characterized as CD45+CD66b+. Mononuclear phagocytes are composed of monocytes and macrophages (16). Immunocyte populations of interest were manually gated as previously reported (17). The viSNE analysis of T cells or glioma-associated microglia/macrophages (GAMs) was performed based on patients with more than 500 cell events in both PBMC and AG tumor lesions. Automatic cluster gate functionality was applied for the hierarchical cluster analysis. R software (version 3.4.0) was used to generate the heatmaps of marker expression or relative marker expression.

### Normalization for Heatmap Data

For **Figures 2E, D** we used log10-scaled values to normalize the data.

For **Figure 3A**, we first calculated the ratio of the value of each GAM cytokine or marker to that of the paired mononuclear phagocytes in PBMCs. Then we log10-scaled the ratio to normalize the values.

### Polychromatic Immunofluorescence Staining

Three AAmut and three AOD samples were collected for polychromatic immunofluorescence staining. Four percent formalin was used to fix the AG specimens and the specimens were embedded in paraffin blocks. For polychromatic immunofluorescence, 3 µm paraffin sections were washed in PBS twice, and permeabilized in 0.2 to 0.5% Triton X-100 (Solarbio). Then the paraffin sections were blocked in 5% normal donkey serum (Jackson Lab) and stained with primary antibody. Fluorescent-conjugated secondary antibodies (ZSGB-Bio) were used to detect the primary antibodies. Fluorescence mounting medium (Dako) was used to mount the sections. As previously described (18), we used the Opal 4-Color Manual IHC Kit (Perkin Elmer) for the analysis of formalin-fixed paraffin-embedded AG sections following the manufacturer’s protocols. Zeiss LSM880 NLO microscope was used to acquire fluorescent images. Primary antibodies were anti-CD45 (OriGene), anti-lba1 (CST), and anti-CD206 (Proteintech). GAMs were defined based on cells that costained with CD45 and lba1. CD206+ GAMs were defined based on cells that were costained with CD45, lba1, and CD206. The percentage of CD206+ GAMs was defined by (CD206+ GAMs)/GAMs.

### Statistical Analysis

For the CyTOF data, five AAmut samples and the paired PBMCs and 10 AOD samples and the paired PBMCs were analyzed. The paired t-test was used to determine significant differences between the AG and paired PBMC samples. The unpaired t-test was used to determine significant differences between the AAmut and AOD lesions. GraphPad Prism software (version 7.00) was used to perform statistical analysis. P values less than 0.05 were considered to be statistically significant.

## Results

### High-Dimensional Single-Cell Immunophenotyping of AG Samples Using CyTOF

**Figure 1:**
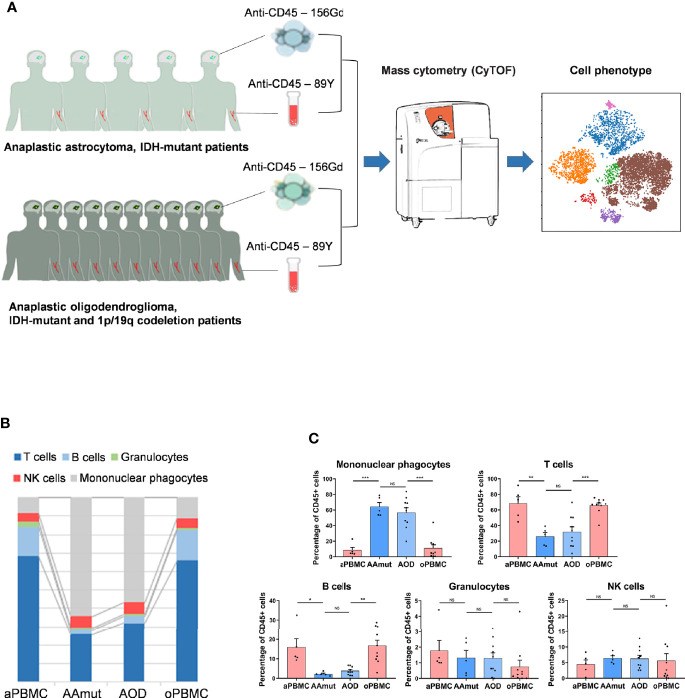
Suppressive immune response to AG tumor lesions. **(A)** Schematics for defining the immune cell composition of AAmuts and AODs. AG tumor lesions and paired PBMC specimens were collected from AG patients. The specimens were prepared and stained with metal isotope-conjugated antibodies. CyTOF single-cell data were analyzed to identify the immune features of the AG patients. **(B)** Constitution of the immunocyte compartment showing the average frequencies of the major immunocyte lineages. **(C)** Bar plots displaying the average frequencies of AG patients and paired PBMC specimens (by paired t-tests and unpaired t-tests). Bar plots show the mean ± SEM (NS, no significance; *p < 0.05; **p < 0.01 and ***p < 0.001).

We obtained 10 AOD tumor tissues, all of which had paired peripheral blood samples (oPBMCs). We also obtained five AAmut tumor tissues and paired PBMC (aPBMC) samples (**Figure 1A**). The baseline characteristics of all AG patients are summarized in **Table 1**.

**Table 1:** Basic characteristics of the AAmut and AOD patients.

Variable	AAmut N = 5	AOD N = 10
Age-mean, years (range)	41.8 (25–61)	45.9 (31–66)
Male	4 (80%)	4 (40%)
Female	1 (20%)	6 (60%)
IDH1		
mutation	5 (100%)	10 (100%)
wild type	0 (0%)	0 (0%)
IDH2		
mutation	0 (0%)	0 (0%)
wild type	5 (100%)	10 (100%)
TERT promoter		
C228T	1 (20%)	7 (70%)
C250T	0 (0%)	0 (0%)
wild type	4 (80%)	3 (30%)
1p19q		
non-codel	5 (100%)	0 (0%)
codel	0 (0%)	10 (100%)

We mapped the immune compartments of the AOD and AAmut lesions and their paired PBMCs at the same time (**Figure 1A**). The initial gating hierarchies for CD45+ immunocytes are demonstrated in **Supplementary Figure S2A**, and **Supplementary Figure S2B** summarizes the gating strategies for the indicated immunocytes. The viSNE map of the CD45+ immunocytes collected from all AG specimens demonstrated differential abundances of infiltrating immunocyte populations in the tumor immune milieu compared to those in the PBMCs (**Supplementary Figure S2C**).

### Mononuclear Phagocytes and T Cells Are the Most Abundant Immunocytes in the AG Immune Microenvironment

We mapped the immune compartment of the AG tumor lesion and the paired peripheral blood specimens at the same time to distinguish the tumor-driven immune changes from the AG immune environment. In the AG immune microenvironment, mononuclear phagocytes (64.16% in AAmut and 56.76% in AOD) and T lymphocytes (25.92% in AAmut and 31.9% in AOD) were the most abundant immunocytes. There were no significant differences in the compartments of immunocytes between the AAmut and AOD immune microenvironments, and the immunocyte compartments in the peripheral blood were also similar. Compared with that in the PBMCs, the ratio of mononuclear phagocytes was notably increased in the AG lesions (p < 0.001 in both AAmuts and AODs), while the ratios of T cells (p < 0.01 in AAmuts and p < 0.001 in AODs) and B cells (p < 0.05 in AAmuts and p < 0.01 in AODs) were notably decreased, and the ratios of NK cells and granulocytes were similar (**Figures 1B, C**).

### T Cells Demonstrate Immunosuppressive Phenotypes in AG

**Figure 2:**
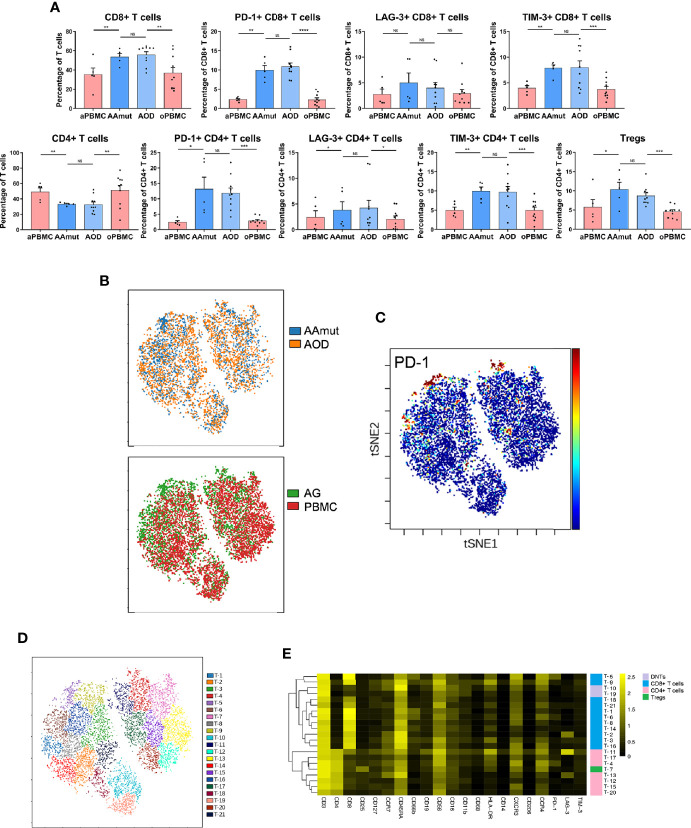
Exhausted T cell compartment in the AAmut and AOD lesions. **(A)** Bar plots displaying the frequencies of the T cell subsets in the AAmut and AOD tumor sites and their paired PBMCs (by paired t-test and unpaired t-test). Bar plots show the mean ± SEM (NS, no significance; *p < 0.05; **p < 0.01; ***p < 0.001 and ****p < 0.0001). **(B)** ViSNE map, colored by specimen source (top) or specimen type (bottom), showing T cell subsets in five AAmut and five AOD patients. **(C)** ViSNE map demonstrating the expression level of PD-1 on T cells. **(D)** ViSNE map displaying T cell subgroups in five AAmut and five AOD patients. The map was colored by clusters. **(E)** Heatmap displaying the normalized indicated marker expression levels for the 21 T cell clusters identified in tumor lesions of the five AAmut and five AOD patients.

Compared with those in the PBMCs, the percentages of CD4+ T cells (p < 0.01 in both AODs and AAmuts) declined, while those of CD8+ T cells (p < 0.01 in both AAmuts and AODs) increased in the AAmuts and AODs. As expected, the proportions of Tregs in the AG lesions were significantly increased in both the AAmuts and AODs (p < 0.05 and p < 0.001, respectively). PD-1-, TIM-3-, or LAG-3- positive T cells are recognized as exhausted subgroups (19–21). Compared to that in the PBMCs, the proportions of PD-1- or TIM-3- positive exhausted T cells were substantially higher at the AG tumor sites (**Figure 2A**).

We employed the viSNE map tool (22) to convert the high-dimensional CyTOF data into a two-dimensional atlas. ViSNE analysis was performed on the patients who gathered more than 500 T cells in both the tumor sites and PBMCs. Finally, five AAmut patients and five AOD patients were analyzed. The viSNE map demonstrates that T cells in the AAmut and AOD groups displayed similar distributions (**Figure 2B**). Compared with the PBMCs, the AG lesions had a certain group of T cells that highly expressed PD-1 (**Figure 2C**).

Based on the hierarchical cluster analysis of the T cells using automatic cluster gate functionality, the T cells were subdivided into 21 subgroups according to the surface markers (**Figure 2D**). The heatmap visualized the expression profiles of these T cell clusters (**Figure 2E**). We identified seven CD4+ phenotypes, eleven CD8+ phenotypes, one Treg phenotype, and two CD4+/ CD8+ double-negative phenotypes with this approach.

### Glioma-Associated Microglia/Macrophages in Anaplastic Astrocytomas Exhibit More Immunosuppressive Characteristics Than Those in Anaplastic Oligodendroglioma

**Figure 3:**
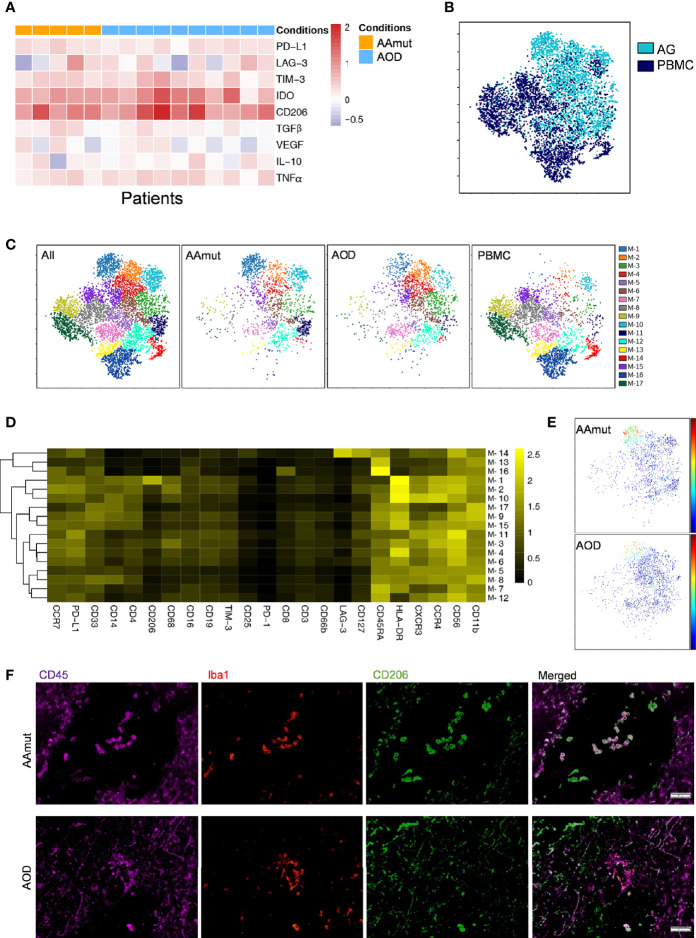
Depiction of the GAM phenotypes in AG. **(A)** Heatmap displaying the relative expression levels of the indicated markers in the 5 AAmut and 10 AOD patients. The relative marker expression levels were defined by the ratios of the indicated marker expression levels of GAMs in AG lesions to those of the mononuclear phagocytes in PBMCs. **(B)** ViSNE map, colored by sample type, demonstrating the GAM subsets in four AAmut and four AOD patients. **(C)** ViSNE map, colored by clusters, demonstrating the GAM subset distribution in the AG tumor sites and the PBMCs. **(D)** Heatmap demonstrating the normalized indicated marker expression levels of the 17 GAM clusters identified in the four AAmut and four AOD patients. **(E)** ViSNE map demonstrating GAMs in AAmut and AOD colored to show the expression level of CD206. **(F)** Representative AAmut and AOD tissues stained for CD45 (purple), lba1 (red), and CD206 (green). Costaining of CD45, lba1, and CD206 (upper) indicated more CD206+ GAMs in AAmut lesions than in ADO lesions. The scale bar corresponds to 50 μm.

Previous studies have shown the strong infiltration of peripheral macrophages and resident microglia within gliomas (23), and macrophages and microglia are collectively termed GAMs. In the current research, GAMs were the most enriched immune cell population in the AG tumor sites compared to the other immune cells. These cells were obviously distinguishable from the mononuclear phagocytes in PBMCs; the GAMs in both AAmut and AOD had higher expression levels of PD-L1, IDO, LAG-3, TIM-3, CD206, and TNFα than mononuclear phagocytes in the PBMCs (**Figure 3A**). Moreover, the GAMs showed intertumoral heterogeneity since CD206, immune checkpoints (PD-L1, TIM-3, and LAG-3), immunosuppressive cytokines (IL-10 and TGFβ), TNFα, and VEGF were expressed at various levels in the AG patients (**Figure 3A**). Compared to the mononuclear phagocytes in the PBMCs, GAMs in AOD lesions expressed higher levels of TGFβ (p < 0.05). Although TGFβ is an immunosuppressive agent (24), there was no significant difference in TGFβ expression levels in GAMs between AAmut and ADO lesions (**Supplementary Figure S3A**).

Four AAmut patients and four AOD patients gathered more than 500 GAM or mononuclear phagocyte event counts in both the AG tumor lesions and the PBMCs, and viSNE analysis was performed based on these cells. The viSNE map demonstrated that the GAMs were obviously distinguishable from the mononuclear phagocytes in PBMCs (**Figure 3B**). With automatic cluster gate functionality, the GAMs or mononuclear phagocytes could be subdivided into 17 subgroups based on the surface markers, and the expression levels of these GAM subpopulations were visualized in a heatmap (**Figures 3C, D**). At the single-cell level, the viSNE map demonstrated a cluster involving M-1, which was described as high expression of CD206, a marker that is expressed by protumor GAMs and may promote a tumor-supportive microenvironment (25). This cluster was excluded from the PBMCs, and there were more CD206+ GAMs in the AAmut lesions than in the AOD lesions (p < 0.05) (**Figure 3E** and **Supplementary Figure S3B**). Polychromatic immunofluorescence confirmed that there were more CD206+ GAMs in the AAmut lesions than in the AOD lesions (p < 0.01) (**Figure 3F** and **Supplementary Figure S3C**).

### NK Cells Act a Complicated Part in the AG Immune Response

**Figure 4:**
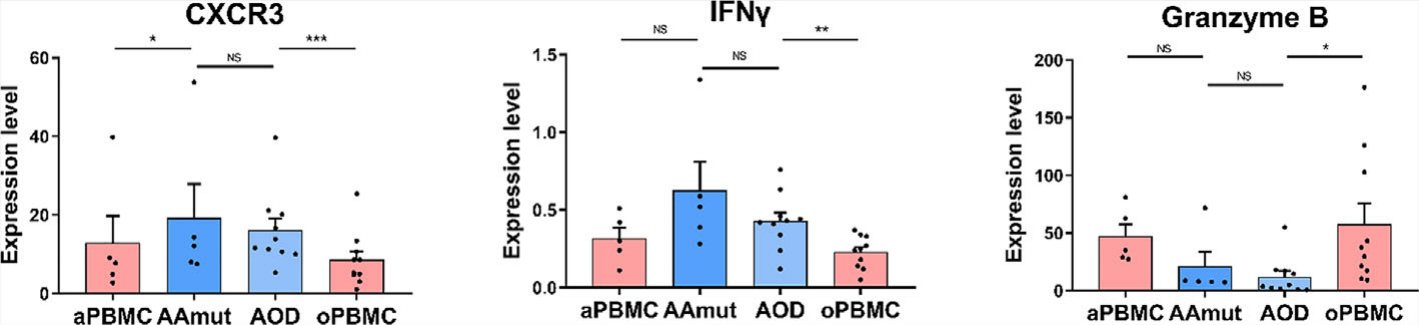
Cytolytic NK cells are dysfunctional at AG lesions. Bar plots demonstrating the expression levels of CXCR3, granzyme B, and IFNγ in the NK cells from AG patients and their paired PBMCs (by paired t-test and unpaired t-test). Bar plots show the mean ± SEM (*p < 0.05; **p < 0.01; ***p < 0.001; and NS, no significance).

It has been reported that CXCR3 is required for NK cell infiltration (26). The expression level of CXCR3 between AAmut and AOD was not significantly different whereas the infiltrated NK cells in the AAmut or AOD lesions expressed higher CXCR3 levels (p < 0.05 or p < 0.001 respectively) than those in their paired PBMCs (**Figure 4**). Although the difference in IFNγ expression level between NK cells in AAmut and ADO lesions was not significant, the NK cells that infiltrated the AOD lesion seemed to demonstrate higher levels of cytolytic activities, as these NK cells expressed higher levels of IFNγ than the paired PBMCs (p < 0.01). Notably, granzyme B expression levels were similar between AAmut and AOD tumor sites, whereas granzyme B expression level was significantly lower in the AOD samples than in the peripheral blood samples (p < 0.05) (**Figure 4**).

## Discussion

In our study, using CyTOF analyses, we analyzed infiltrating immunocytes from surgically resected initial AG tissues, including AAmut and AOD samples. Based on a panel of 33 markers, we present a single-cell view of the complicated AAmut and AOD immune microenvironment. Our study verified that mononuclear phagocytes and T cells were the most abundant groups in the immune microenvironment of AGs. The GAMs in both AAmut and AOD showed substantial inter- and intratumoral heterogeneity with highly immunosuppressive characteristics. Compared to that in the PBMCs, the ratios of immune checkpoint-positive exhausted CD4+ T cells and CD8+ T cells were distinctly higher at the AG tumor sites. The immune microenvironment in AAmut exhibits more immunosuppressive characteristics than that in AOD.

AGs are regarded as intermediate-grade gliomas, whose malignancy is between low-grade gliomas and glioblastomas (GBMs) (27). Furthermore, AGs are infiltrative neoplasms with a highly invasive nature, in which the disruption of the blood-brain barrier (BBB) is between that of low-grade gliomas and GBMs (28). Both the malignancy of the tumor and the breakdown of the BBB contribute to the unique and specific immune microenvironment in low-grade gliomas and GBMs (8). In our study, mononuclear phagocytes and T cells were the most abundant groups in the immune microenvironment of the AGs. The GAMs in the AGs present highly with various immunosuppressive cytokines and chemokines among the patients and among GAM subsets. Compared to those in the PBMCs, the ratios of exhausted CD4+ T cells and CD8+ T cells were distinctly higher at the AG tumor lesions. CyTOF technology provides a high-dimensional view of the composition of the immunosuppressive microenvironment in AGs, which may be vital for effectively targeting the immunosuppressive subpopulation in a clinical setting and for the special design of future immunomodulators for AGs.

It has been reported that the prognosis between WHO grade III AAmuts and AODs is significantly different, and AOD patients have a better overall survival time than AAmuts patients (7). Glioma cells promote the infiltration of a range of immune cells into the tumor site by secreting numerous cytokines, chemokines, and growth factors (29–31); these nonneoplastic elements create a specific niche called an immune microenvironment. The immune microenvironment plays a vital role in the glioma response to treatment and prognosis (8, 32, 33). A systematic view of the immune milieu that differs between AAmuts and AODs is still lacking. Using the CyTOF method, on a single-cell basis, we showed that GAM clusters in AAmut were characterized by higher expression of CD206 than those in AOD. CD206 is a prominent prognostic marker that is specifically expressed by protumor GAMs, meanwhile the immune milieu plays a vital role in glioma progression and prognosis (8, 25). Our results implied that immunocytes especially GAMs in AAmut exhibit more immunosuppressive characteristics than those in AOD and that the different immune microenvironments of AOD and AAmuts might be partial reasons for their different prognoses.

Granzyme B has been traditionally viewed as a primary mechanism used by NK cells to eliminate tumor cells (34). NK cells in both AAmut and AOD lesions expressed lower levels of granzyme B than those in PBMCs. Meanwhile granzyme B in NK cells between AAmut and ADO lesions demonstrated similar expression levels. This suggests that in the AG immune microenvironment NK cells might act a complicated part in the immune response which needs further exploration.

Our study has several limitations. The IDH status of glioma was shown to affect the tumor immune state and progression (35, 36). Deciphering the immune milieu of AGs and clarifying the differences between AAmuts and AAwts and patient prognosis requires further research and the small number of cases may not be enough to identify the immune microenvironment differences between AAmuts and AODs, which needs the collection of more cases and further exploration. Although CyTOF makes the concurrent measurement of more than 30 parameters per single cell possible (37), the limited number of surface markers measured simultaneously still restricts the analysis. In-depth studies such as single-cell RNA sequencing are needed to further validate our findings.

## Data Availability Statement

The CyTOF data used and analyzed in this study are accessible from https://premium.cytobank.org/cytobank/experiments/332858.

## Ethics Statement

This study was approved by the Institutional Review Board and Ethics Committee of Beijing Tiantan Hospital, Capital Medical University. All patients provided written informed consent.

## Author Contributions

YC, DC, JFW, SW and JZ conceived and designed the study. WF and WW analyzed and interpreted the CyTOF data. HL, YJ, RH, JW, JCW, HX, and ZY participated in sample collection and data acquisition. All authors contributed to the article and approved the submitted version.

## Funding

The current research was supported by the “Beijing Scholar Program 2015”.

## Conflict of Interest

The authors declare that the research was conducted in the absence of any commercial or financial relationships that could be construed as a potential conflict of interest.

## Supplementary Material

Supplementary Figure 1 Mass cytometry panel. Markers used to characterize AAmut and AOD immune phenotypes.

Supplementary Figure 2 Analysis of the immune microenvironment of AG using CyTOF. **(A)** Gating hierarchy for identifying the CD45+ cells. a) EQ3 beads and EQ4 beads were utilized to recognize cell events from all events. b) Single living cells were recognized by gating the cell events positive for 193Ir and negative for 195Pt. c) CD45+ cells from AGs and PBMCs were gated from the single living cells. **(B)** Cell type identification strategies. **(C)** ViSNE plots of the immunocytes in all samples based on the relative expression levels of the CyTOF markers. The cell colors show the expression level of the indicated markers. The immunocyte populations are indicated as well (left). Five hundred CD45+ immunocytes per specimen were included in the viSNE analysis.

Supplementary Figure 3 Comparison of the immune microenvironment between AAmut and AOD samples. **(A)** Bar plots displaying TGFβ expression from GAMs or mononuclear phagocytes in the AAmut and AOD tumor sites and their paired PBMCs (by paired t-test and unpaired t-test). Bar plots show the mean ± SEM (*p < 0.05 and NS, no significance). **(B)** Bar plots displaying the frequencies of the CD206+ GAMs the AAmut and AOD tumor sites using CyTOF (by unpaired t-test). Bar plots show the mean ± SEM (*p < 0.05). **(C)** Bar plots displaying the frequencies of the CD206+ GAMs the AAmut and AOD tumor sites using polychromatic immunofluorescence staining (by unpaired t-test). Bar plots show the mean ± SEM (**p < 0.01).
