# Supplementary figures and images for: CyTOF Analysis Reveals a Distinct Immunosuppressive Microenvironment in IDH Mutant Anaplastic Gliomas
Source: Front Oncol. 2021 Feb 4;10:560211. doi: 10.3389/fonc.2020.560211 (PMC7890006; doi:10.3389/fonc.2020.560211)

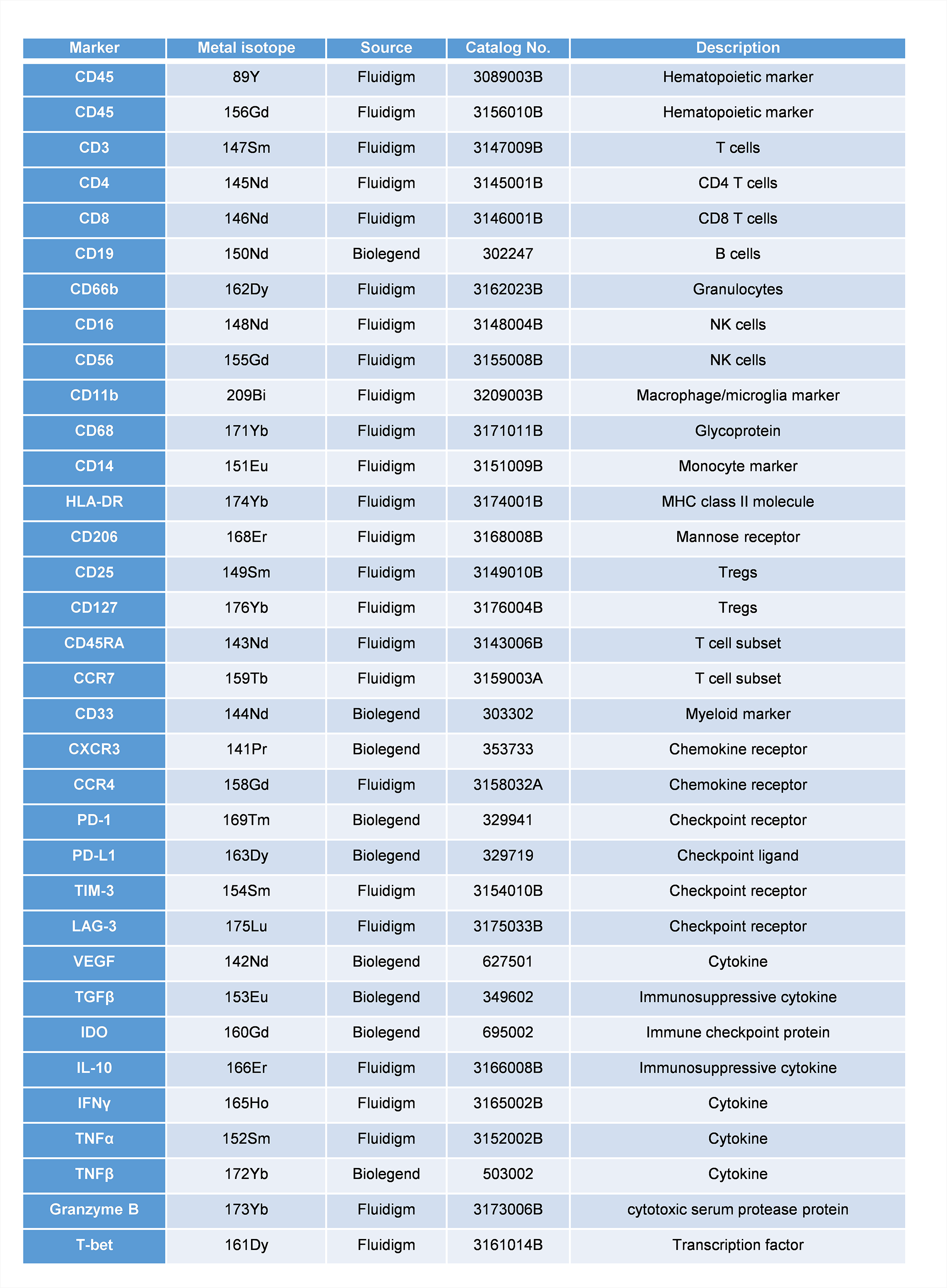

Supplement: Supplementary Figure 1 — Mass cytometry panel. Markers used to characterize AAmut and AOD immune phenotypes. [file Image_1.tif]

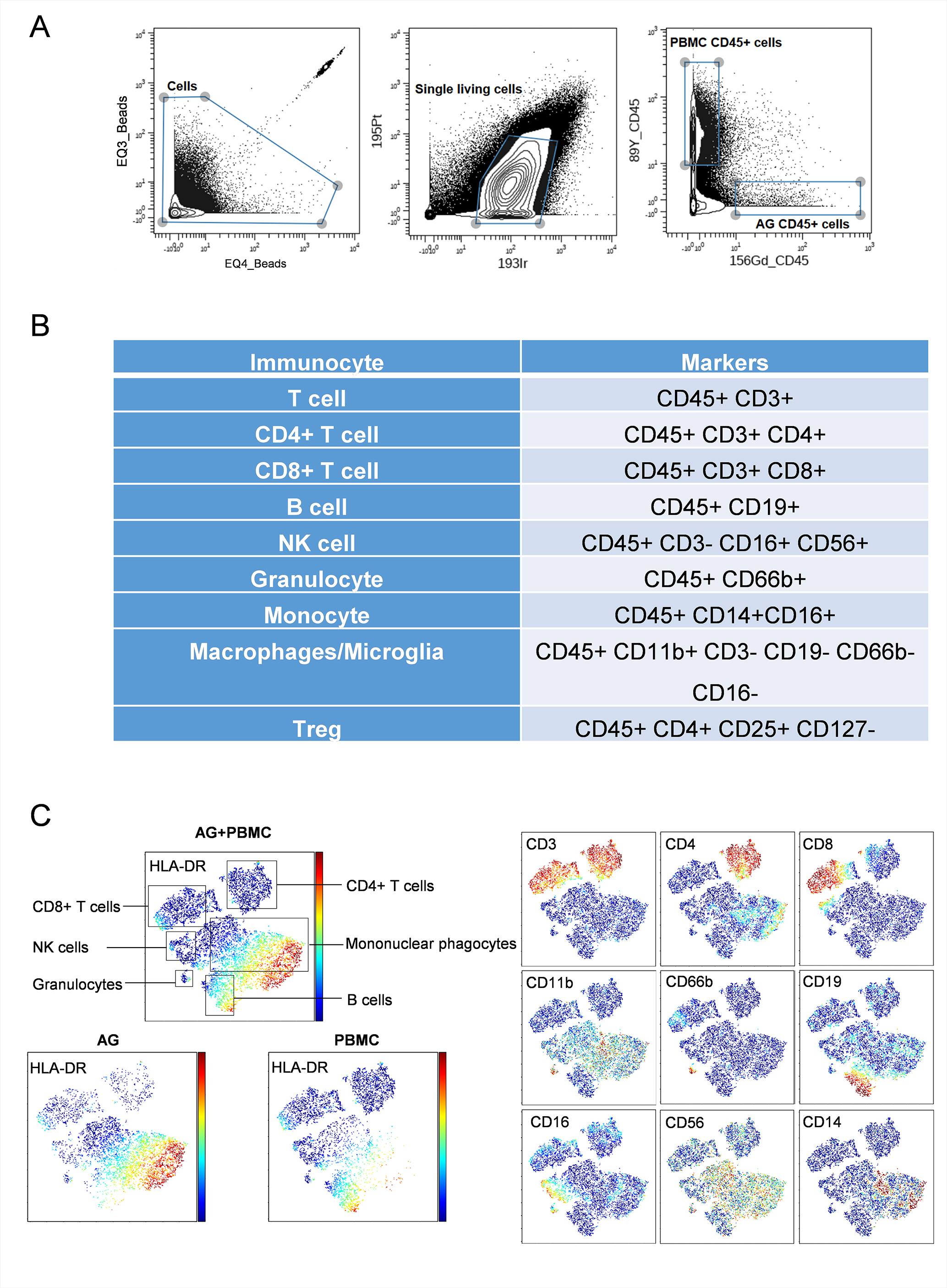

Supplement: Supplementary Figure 2 — Analysis of the immune microenvironment of AG using CyTOF. (A) Gating hierarchy for identifying the CD45+ cells. a) EQ3 beads and EQ4 beads were utilized to recognize cell events from all events. b) Single living cells were recognized by gating the cell events positive for 193Ir and negative for 195Pt. c) CD45+ cells from AGs and PBMCs were gated from the single living cells. (B) Cell type identification strategies. (C) ViSNE plots of the immunocytes in all samples based on the relative expression levels of the CyTOF markers. The cell colors show the expression level of the indicated markers. The immunocyte populations are indicated as well (left). Five hundred CD45+ immunocytes per specimen were included in the viSNE analysis. [file Image_2.tif]

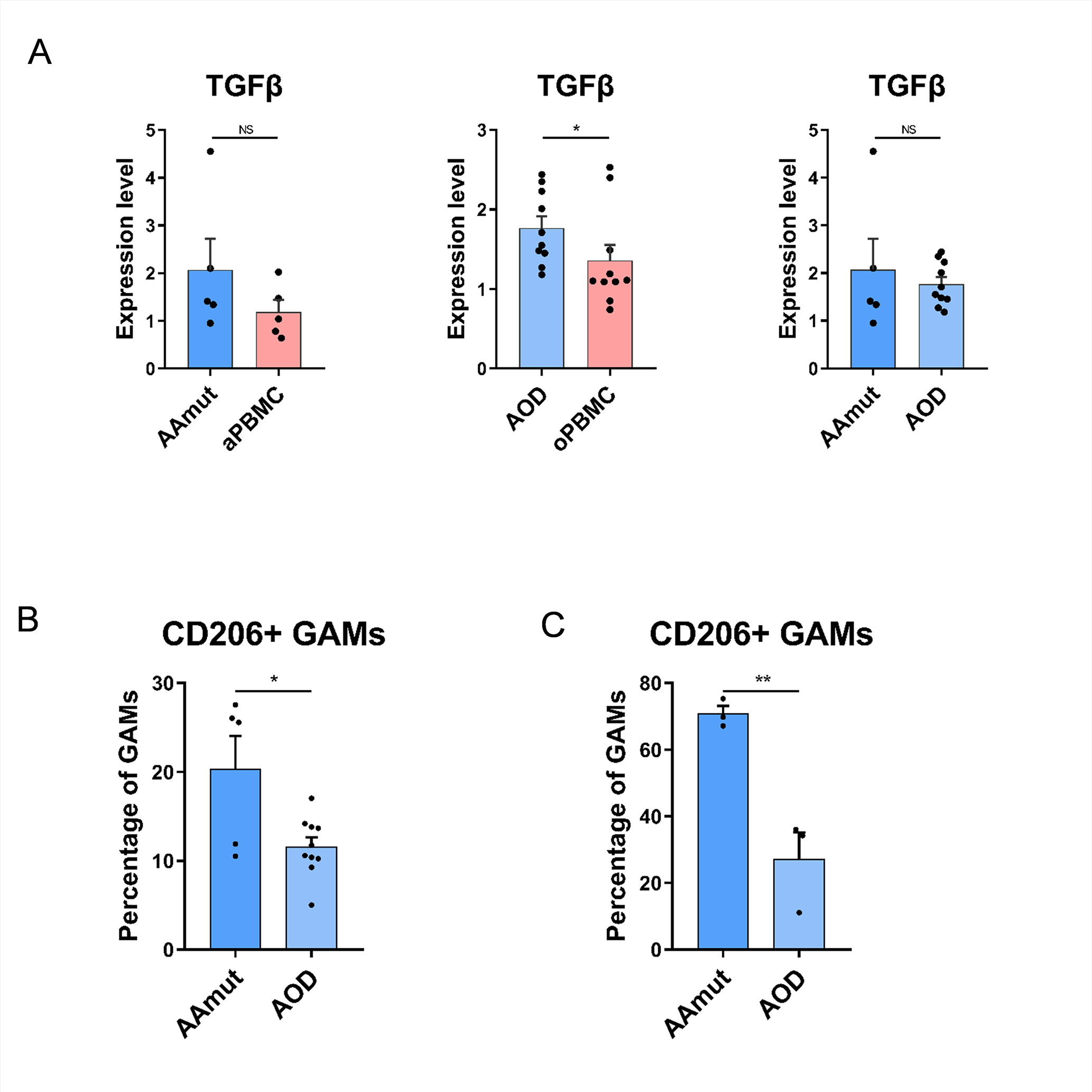

Supplement: Supplementary Figure 3 — Comparison of the immune microenvironment between AAmut and AOD samples. (A) Bar plots displaying TGFβ expression from GAMs or mononuclear phagocytes in the AAmut and AOD tumor sites and their paired PBMCs (by paired t-test and unpaired t-test). Bar plots show the mean ± SEM (*p < 0.05 and NS, no significance). (B) Bar plots displaying the frequencies of the CD206+ GAMs the AAmut and AOD tumor sites using CyTOF (by unpaired t-test). Bar plots show the mean ± SEM (*p < 0.05). (C) Bar plots displaying the frequencies of the CD206+ GAMs the AAmut and AOD tumor sites using polychromatic immunofluorescence staining (by unpaired t-test). Bar plots show the mean ± SEM (**p < 0.01). [file Image_3.tif]
